# Social Identity, Perceived Emotional Synchrony, Creativity, Social Representations, and Participation in Social Movements: The Case of the 2019 Chilean Populist Protests

**DOI:** 10.3389/fpsyg.2021.764434

**Published:** 2021-12-09

**Authors:** Pablo Castro-Abril, Silvia Da Costa, Ginés Navarro-Carrillo, Angélica Caicedo-Moreno, Marcela Gracia-Leiva, Pierre Bouchat, Begoña Cordero, Lander Méndez, Darío Paez

**Affiliations:** ^1^Department of Social Psychology, Faculty of Psychology, University of the Basque Country UPV/EHU, Donostia-San Sebastian, Spain; ^2^Faculty of Social and Human Sciences, University of Zaragoza, Teruel, Spain; ^3^Department of Psychology, Faculty of Humanities and Education Sciences, University of Jaén, Jaén, Spain; ^4^Department of Psychology, Université de Lorraine, Metz, France; ^5^Faculty of Psychology, University of Talca, Talca, Chile

**Keywords:** populist social movement, social identity, perceived emotional synchrony, creativity, Chile, collective action and social movements

## Abstract

This paper analyzes the socio-cognitive and emotional processes related to collective action in the context of the 2019 populist social movement in Chile. It proposes an integrative explanation of populism as social movements and collective gatherings along with their relation with creativity and social representations of mass movements. A comprehensive online survey was used (*n* = 262) that included measures of participation in demonstrations, identification with protesters or the government, agreement with social movement grievances, collective efficacy, perceived emotional synchrony, collective action, self-reported cognitive creativity, and individuals’ proposals for improvement of society and ideas associated with stimuli (e.g., the concepts of majority or minority). Our results revealed that identification with demonstrators, agreement with protesters’ grievances, a high perceived emotional synchrony or collective effervescence, and higher creativity responses were associated with an active participation in the social movement. Higher participation and factors conducive to participation were associated with lexical clusters of responses to stimuli that include words such as rights, justice, injustice, bravery, dignity, or hope, which were conceived of as positive social representations of the populist social movement. These findings are discussed within the neo-Durkheimian framework of collective gatherings and the perspective of populism as a social movement that seeks to renew and expand democracy.

## Introduction

Since October 17, 2019, Chile has been undergoing a social movement of an unprecedented scale. This movement, born as a reaction to increases in the prices of public services, quickly evolved into a denunciation of the huge economic and social inequalities of Chilean society that have been dragging on for decades. An acephalous nature and low level of political party influence characterize the movement. Despite the withdrawal of a state of emergency declaration and the adoption of several social measures, protests continued for several months. Even 2 years later, the social movement is still active and successfully imposing a Constituent Assembly. This study aims to highlight the psychosocial factors at work within this populist social movement, emphasizing the role played by group emotion-related processes in collective action. Populist social movements can be seen as non-institutional collective mobilization along a catch-all political platform of grievances that divides society between an overwhelming majority of “pure people” and a “corrupt elite,” demanding the restoration of popular sovereignty in the name of the former (Aslanidis, 2017). Populism is conceived as set of attitudes and beliefs or ideology, a rhetoric or social communication style, and a political and social movement emphasizing that the people are in a morally charged battle against the elites (Mansbridge and Macedo, 2019; Noury and Roland, 2020, p. 60)^1^.

Spontaneous “anti-austerity protests” in Latin America, such as the ones in Chile during 2019–2020, are “social movements in opposition to what is perceived as the illegitimate dismantling of a historically negotiated contract between state and people; such movements are, however, political in a broader sense—with their central concern for social justice and the moral economy, they constitute a distinctive form of populist movement” (Walton and Seddon, 1994; see Aslanidis, 2017 for a broader discussion). It is important to highlight the conditions that describe the Chilean social outbreak and correspond to the attributes seen in different experiences of populism in Latin America (Bonnin, 2020). First, the movement arose from a situation of crisis or change as described before. Second, an approach was used that prioritizes participatory democracy over representative democracy as seen in the petitions for the plebiscite for a new constitution. Third, the movement is framed in a context of intrinsic historical ambiguity in which Chile has traditionally been seen as an economic example of the region while levels of inequality and poverty increase for most of the population (Flores et al., 2020). Moreover, these social protests reflect a crisis of institutionalized, party-mediated democratic representation, which generates the political opportunity for an anti-government discourse that has been persistent in the movement (e.g., demanding the president’s resignation; Roberts, 2017). On the other hand, the rapid evolution of the social movement managed to agglutinate a large number of demands, groups, and social collectives, seeking to represent the “people,” understood as a collective entity that transcends individuals and draws on anti-liberal political traditions. Although populism is used as a pejorative term (Rovira et al., 2017), populist movements in Latin America are related to contradictory processes; notoriously, the process of domination, but also of liberation and agency, due to the conception of “the people” as a complex social identity rather than a pre-existent demographic entity (Bonnin, 2020). In this regard, populist movements such as the Chilean one differ from other populist political and social movements in four aspects: (a) Their quest is to represent a total collectivity as opposed to a particular social group; (b) Their objective is to make major structural and political changes that address the issues causing the crisis (Aslanidis, 2017); (c) They lack the need for a populist leader due to the weight given to “the people” as a social identity (Bonnin, 2020); (d) They aim to expand democracy (Mansbridge and Macedo, 2019) and do not associate themselves with authoritarian restrictions of democracy or support for strong “Bonapartist” government leadership (e.g., the governments of Peron, Vargas, or Ibañez in Argentina, Brazil, or Chile).

In the last decades, collective action has become a central topic in the social sciences and their integration with populism studies was requested. In particular, the study of bottom-up populist mobilization or populist social movements can bring together theories of populism with models of social mobilization (Aslanidis, 2017). Through three meta-analyses, van Zomeren et al. (2008) identified three main predictors of collective action: (a) perceived injustice (e.g., “It is only when social comparisons lead to a subjective sense of injustice that collective action to redress injustice is likely to occur,” p. 505); (b) social identity, defined as a subjective sense of identification with a group (e.g., “If group members perceive intergroup status differential to be illegitimate and unstable, they are more likely to identify with their group and engage in collective action to change the intergroup status differential,” p. 507); and (c) perceived efficacy (“People engage in collective action if people believe this will make it more likely that relevant goals are achieved,” p. 506). Among these predictors, the affective experience of injustice, moral conviction, and a politicized social identity have stronger effects on collective action than non-affective perceived injustice and non-politicized identity (van Zomeren et al., 2012). This model echoes populist mobilization based on the following factors: (a) Felt injustice, building a social movement upon the conviction that individuals invested with political authority (Piñera’s government and political parties) are deliberately falling short of serving the needs of the people they were supposed to represent. Populist grassroots movements fulfill an aggregative function as a “mode of articulation” of social grievance, group deprivation and injustice; (b) Populist social movements frame a collective action of social actors (e.g., Chilean people) sharing a principle of identity, in a conflictive social relationship with a collective adversary who dominates and rules (e.g., ineffective and corrupt political elite). Constructing collective identities is a crucial function of social movement activism, and populism constitutes an exemplary case of identity mobilization under the inclusive banner of “the people” against the inefficient and corrupt political caste or elite; and (c) Finally, in a globalizing world where party systems are being rendered unable to function as channels for the expression of popular demands, populist movements have become an appeal to exercise bottom-up underdog agency, recovering sovereignty from powerful elite minorities (Aslanidis, 2017).

In addition to perceived injustice and/or grievances, social and collective identity, and perceived efficacy, recent studies have stressed the role played by group emotions in collective action. From that perspective, emotions are considered amplifiers of existing motivations. Anger and moral indignation can a bridge sensitivity to social issues and collective action and have characterized many grassroots populist social movements: Indignados and Occupy Wall Street, the Arab Winter uprising, and the recent protests in Colombia, Ecuador, Peru, and Chile (Aslanidis, 2017; Wlodarczyk et al., 2020). In the present paper, we will expand on this perspective by considering socioemotional processes related to collective gatherings, demonstrations, and populist collective action.

### Microsocial Processes Explaining the Emergence of a Social Movement

Above, we presented the main predictive factors of participation in collective action and populist grassroots social movements, and pointed to the role of social identity, perceived injustice, perceived efficacy, and emotions. But how do individuals and groups build or rebuild and strengthen a collective identity, build a vision of grievances, and identify those responsible for them and how they build a feeling of collective efficacy? It is important to remark that studies support the notion that participation in collective gatherings and demonstrations (Rimé et al., 2010; Páez et al., 2015; Vestergren et al., 2016) strengthens collective identity and empowers participants, increasing collective self-esteem and efficacy, positive emotions, and social beliefs.

This text examines two mechanisms that account for the positive social psychological effects of collective gatherings on the predictive factors of social mobilization and participation in collective action. The first focuses on the socio-cognitive process of self-categorization, and the second points to the role of perceived emotional synchrony during the gatherings.

### Socio-Cognitive Processes Related to Collective Identity

From the perspective of self-categorization theory, emphasis is placed on participation in demonstrations, the contextual experience of interaction with law enforcement forces, and the socio-cognitive processes of social categorization that lead to collective identification. According to Drury and Reicher (2005), the validation of a specific collective identity during collective actions, or a sense of shared identity that is developed among members of a demonstration, is the main process that leads to the strengthening of participation in the movement (Novelli et al., 2013; Hopkins et al., 2016). In line with this approach and given the Chilean context of defiance toward the elites and repression, we formulate the following hypothesis:

(1) Participation in demonstrations, and repertoire of participation, should be related to collective identity-related factors, such as social identification with demonstrators and disidentification with the elite and the government, agreement with movement’s grievances, and collective efficacy.

### Socio-Emotional Processes Related to Collective Effervescence

The socio-cognitive approach of collective action has been supported by numerous empirical results and has extended beyond the framework of social psychology (Dixon et al., 2020). Still, it makes little sense of the emotional experience in the understanding of the effects of collective gatherings on collective action. A socioemotional approach complements the cognitive self-categorization (Livingstone et al., 2011) and extends to those social instances where there is not a prior sense of shared identity, and yet can result in sustainable forms of it (Páez et al., 2015). In this approach, collective effervescence during participation (direct or mediated by mass and social media) is considered another important explanatory mechanism of involvement in collective action. Durkheim (1912/2001) described collective effervescence as the intensification of emotions by social sharing and a state of high arousal that takes place in religious and secular rituals. Collective effervescence can also be conceived as exuberant reactions specific to this type of emotional episode that empowers individuals, enhancing individual and collective esteem and efficacy (Collins, 2004; Wlodarczyk et al., 2020). Recent empirical evidence supports his view and shows how this mechanism of collective effervescence or perceived emotional synchrony^2^ fuels a series of positive psychosocial consequences, such as increases in collective identity, social cohesion, positive and self-transcendent emotions, adhesion to values, and pro-social beliefs (Hopkins et al., 2016; Pelletier, 2018; Bouchat et al., 2020; Wlodarczyk et al., 2020). In line with the Durkheimian approach to collective gatherings, we formulate the following hypothesis:

(2) The level of perceived emotional synchrony felt in recent demonstrations (i.e., collective effervescence felt during participation or when exposed to social and mass media display of demonstrations) should positively predict the engagement in a large repertoire of forms of collective action and beliefs related to the mobilizations controlling for collective identity and sociodemographic variables.

**FIGURE 1:**
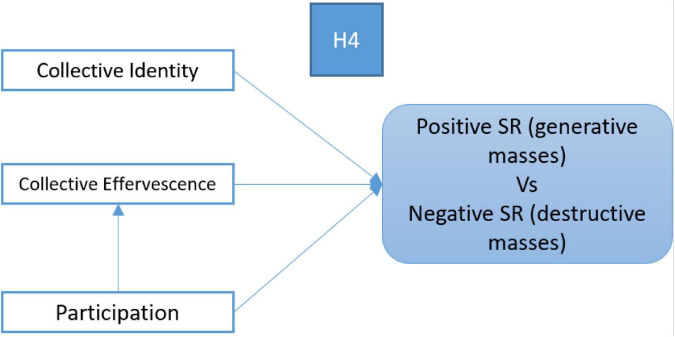
Behavioral (participation), socioemotional predictors (collective effervescence), and socioemotional variables as positive predictors of generative and benevolent Social Representations of Masses and negative predictors of barbarian Social Representations of masses.

The following figure expose these hypotheses (see Figure 1).

### Collective Effervescence and Creativity

**FIGURE 2:**
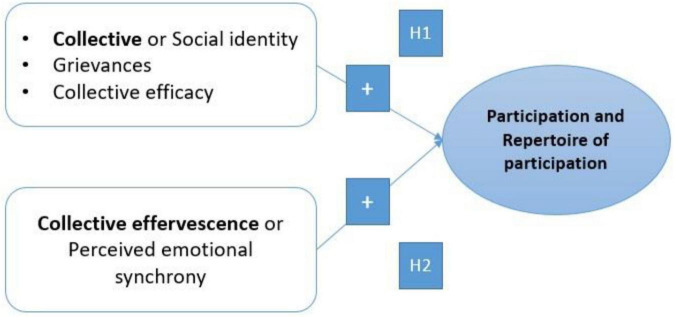
Socio-cognitive and emotional predictors of quantitative (participation versus non-participation) and qualitative (large repertoire of actions) social mobilization or participation.

Another aspect of Durkheim’s conception of collective gatherings relates to their impact on social creativity. Durkheim observed that collective gatherings and related emotional effervescence potentially elicit creativity and aid in creation of new symbols and ideologies. Creativity is defined as the creation of new, different, and useful ideas (Amabile, 1988; Averill, 2005; da Costa et al., 2015). In his seminal book on religious rituals, Durkheim (1912/2001, p. 158) argued that “there are periods in history when, under the influence of some great collective shock, social interactions have become much more frequent and active…. That general effervescence result is characteristic of revolutionary or creative epochs.” Examining the social mobilization consequent to the Dreyfus affair, Durkheim observed that “people took the streets, flags were waved, creeds professed, ideals renewed. In the midst of such effervescence, the sociologist discerned a common faith (civic republicanism) reaffirmed and extended” (Cladis, 2001, p. 15). Two meta-analyses found that high arousal positive affect is positively associated with creativity (da Costa et al., 2015). Collective effervescence as an emotional context should evoke high arousal positive affect, which in turn would engender a greater number of ideas and reinforce originality or the generation of more uncommon ideas, because the associative network of emotional states and positive materials promotes memory and accessibility of information. Furthermore, high arousal positive affect also plays the role of a signal, suggesting that a state of well-being prevails, evoking a divergent thinking style, and boosting attention and the repertoire of actions and ideas (Fredrickson, 2013). Hence, this study also explores the creative responses of people experiencing social mobilization, understanding that in the case of Chile, social mobilization requires the search for solutions and the demand for new ways of conducting politics in the country. The creative experience can also benefit from the emerging social movement, from the cognitive and emotional route that we propose. Following these observations, we formulate a third hypothesis:

(3) Perceived emotional synchrony should positively predict cognitive creativity controlling for sociodemographic and ideological variables, along with participation in demonstrations H3a (see Figure 2). Moreover, we further postulate that perceived emotional synchrony will play a mediating role between participation in demonstrations and creativity H3b.

### Social Representations of Mass Movements

Social representations are common sense beliefs that assimilate and reproduce more elaborated discourse. Two important discourses are important with respect to spontaneous collective gatherings or demonstrations related to social movements. While one emphasizes the negative aspects of populist movements (e.g., perceiving crowds as “barbarian,” violent, and destructive; McPhail, 1997), the second discourse (based on Durkheim) conceives collective gatherings and popular movements as moments of creativity, enhancing well-being, social cohesion, and moralization through renewed agreement upon values and ideal social beliefs (Moscovici, 1988; Drury, 2014). Nonetheless, both approaches share the ideas of intense emotionality, convergence, and polarization of opinions, along with emotions and behaviors during participation in mass movements (Moscovici, 1988).

**FIGURE 3:**
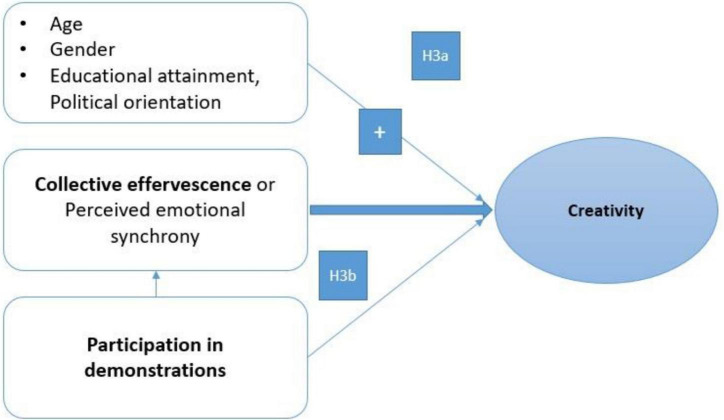
Predictors of creative performance.

The view of a “barbarian, crazy, violent” crowd of demonstrators and its emphasis in “restoring social order” are potential central contents of a social representation for people with an unfavorable attitude and who are reluctant to participate in collective behavior. The opposite perspective is a “Durkheimian” view of demonstrations as instances of creation of new ideas and values that guide social change relative to social identification and participation in demonstrations (Moscovici, 1988; Drury, 2014). We formulate the following as our fourth and last hypothesis:

(4) Positive social representations (e.g., a just fight opposing injustice, new ideals, and social change) or clusters of beliefs about demonstrators and a populist movement would be associated with higher level of participation and factors conducive to social identification with demonstrators, collective efficacy, and perceived emotional synchrony. In contrast, a negative social representation of a populist social movement (e.g., demonstrations as violent, non-rational crowd behaviors) should be associated with non-participation and low scores in factors conducive to identification with demonstrators, etc. Figure 3 depicts this hypothesis.

In this empirical research, words written in association with images that come to mind when thinking about demonstrators, the police, responses to stimuli, majority, minority, and inequality, and a description of demonstrations as a reminder of them will be analyzed using the Iramuteq program. We will examine how these conceptions are associated with responses associated with participation in demonstrations, factors conducive to participation in demonstrations, and political position. In this sense, structures of meaning will be articulated with participation in demonstrations and psychosocial factors, examining whether these social representations are anchored in social position and attitudes.

### The Chilean Context

In October 2019, a social movement arose as a reaction to increases in the prices of public services. It then quickly evolved toward a denunciation of the huge economic and social inequalities of Chilean society. Soon after the outbreak of the protest movement, pacific demonstrations were harshly repressed, reinforcing a sense of injustice and provoking a delegitimization of law enforcement forces and government. Surveys carried out in 2019 and 2020 indicate 83% of disapproval of President Piñera, in contrast to 53% in 2018 (Amnesty International, 2019; Activa, 2020; Criteria, 2020). Indeed, the brutal police repression and governmental mismanagement deepened the crisis once it exploded (Somma et al., 2021). The repression targeted both those demonstrating violent acts and peaceful protesters, causing severe injuries in some cases (Human Rights Watch, 2020). After the first demonstrations and police repression, large mobilizations emerged and an emotional atmosphere of outrage, anger (42%), and joy/hope (43%) appeared (Feedback, 2019)^3^. Despite the imposition of a state of emergency and the presence of the army in the streets, people keep demonstrating (Amnesty International, 2019; United Nations Human Rights, 2019; Human Rights Watch, 2020). Around half of the young adult population participated in the October demonstrations (40%) and protested using pots as drums (52%)^4^. The social protests showed a confluence of urban citizens, Mapuche and non-Mapuche actors, and rural communities. The demands for more social dignity and social rights in protest coincide with the struggle of the Mapuche people, who have suffered extensive abuses and human rights violations from the state for demanding collective and territorial rights for decades (e.g., murder of Camilo Catrillanca by the police, November 2018) (Pedemonte and Gálvez, 2021).

Within a few weeks, reaction to government inactivity and police repression brought more than 1,200,000 people to the streets of the main cities of the country (Villarroel and Flores, 2019).^5^ Regarding creativity, in the Chilean case, demonstrations began with an innovative way of protesting (jumping over the subway turnstile), a lot of slogans were generated, places and monuments were renamed, and the main square of Santiago was called the Square of Dignity. In demonstrations, it was common to see the indigenous Andean (wiphala) and Mapuche (wenufoye and wünelfe) flags, emblems, and posters (Somma et al., 2021) used by both indigenous and non-indigenous protesters as counter-hegemonic symbols (Blair, 2019). It highlighted the symbolic and political weight linked to the Mapuche social movement. Innovative performances, such as Las Tesis’s feminist action, were largely replayed across the country—50% of a representative sample knows this feminist performance, and 80% have a positive opinion about it (Cadem, 2020). On the other hand, the 2019 demonstrations have been considered the “worst civil unrest” in Chile since the end of military dictatorship due to the scale of damage to public infrastructure, the number of protesters, and the repressive measures taken by the government. Although it is true that people destroyed buildings and facilities and that clashes with the militarized police were frequent, the deaths of people caused by the demonstrators were non-existent. Police repression, however, did cause several deaths and the total or partial blindness of two hundred demonstrators. Similarly, property owners defending their business from looting had also caused injuries to civilians. Not a single police officer was killed by protesters (National Institute of Human Rights, 2020). Mobilization persists in 2020–2021, despite the COVID-19 pandemic, although it decreased due to the confinement measures. More importantly, the call for a constituent assembly was approved with a strong presence of protest leaders elected as representatives.

## Materials and Methods

### Participants and Procedure

We tested our hypotheses in an online questionnaire, which was distributed to students and former students of private universities in two big cities in Chile. The distribution method was through e-mails and more than two thousand e-mails were sent. With the Qualtrics Survey Platform^®^, online surveys were prepared and accessed *via* a link. The response rate was around 20%, which is common in this kind of data collection (Shih and Fan, 2009). Between October 23 and November 30 (2019), a total of 262 participants (64% women; Mage = 35.90, *SD* = 12.51) completed the questionnaire, which was presented to them in their language of education (i.e., Spanish)^6^. The timeframe for data collection was established based on the main demonstrations and events during the social movement. Massive protests took place during the first weeks of October, and by October 19, the president declared a state of emergency that heavily restricted mobility and social gatherings. Due to the large scale of the social unrest seen throughout this period, the media had an almost uninterrupted coverage of the demonstrations, governments’ actions, and other related events (Garcés, 2020). Our interest remained in collecting as much information as possible as the main events of the social movement occurred to properly evaluate the effect of collective action on the selected variables. A total of 61% had finished graduate studies and 33% postgraduate studies. Completing the full questionnaire took, on average, 10–15 min. All study participants read and accepted informed consent. The data recorded was alphanumerically code to ensure anonymity following the Organic Law on the Protection of Personal Data (BOE-A-2018-16673), and compliance with the regulation of the Ethics Committee for Research Involving Human Beings (CEISH) by the University of the Basque Country.

### Instruments

#### Past Reported Collective Behavior

##### Participation in Demonstrations

To determine whether respondents participated in the mobilizations, a dichotomous (no = 1, yes = 2) measure (“Did you participate in the demonstrations?”) was used. A total of 71% of respondents reported having participated in demonstrations.

#### Socio-Cognitive or Collective Identity-Related Factors

##### Social Identification

We evaluated the extent to which respondents identified with demonstrators and the government by administering two items (e.g., “*What is your level of identification with the demonstrators?”*). Responses were given on a Likert scale ranging from 0 (*not at all*) to 7 (*very much*). These items were negatively correlated (*r* = –0.62, *p* < 0.001). In addition, we calculated an index of identification with demonstrators and disidentification with the elite or government by subtracting respondents’ levels of identification with the government from the respondents’ scores on identification with protesters.

##### Agreement With Protesters’ Grievances

We asked participants to indicate their levels of agreement with the grievances linked to the social mobilizations by using a three-item measure (e.g., “What level of agreement do you have with the public protests/demonstrations?”). It has a 5-point Likert response format ranging from 1 (totally disagree) to 5 (totally agree). Reliability was adequate (α = 0.71).

##### Collective Efficacy

Participants’ levels of perceived group efficacy regarding the protests were assessed by including the Collective Efficacy (CE) scale targeted at Chilean people, a short four-item version adapted from the CEQS-Collective Efficacy Questionnaire for Sports (Martínez et al., 2011). This measure (e.g., “I believe we are able to reach our goals”) has a 6-point Likert response format ranging from 0 (not at all) to 5 (very much). Reliability was satisfactory (α = 0.87).

#### Socio-Emotional Factors

##### Collective Effervescence

A shortened version of the Perceived Emotional Synchrony Scale (PESC) developed by Wlodarczyk et al. (2020) comprising a total of six items was administered to evaluate respondents’ levels of emotional effervescence experienced with regard to the protests (e.g., “We felt that we were one”). Responses were given on a Likert scale ranging from 1 (not at all) to 7 (very much). Reliability was very satisfactory (α = 0.92).

#### Collective Action Variables

##### Repertoire of Participation

Different forms of engaging in the social protests/mobilizations were evaluated by means of a five-item measure [e.g., “I have manifested myself through pots and pans (cacerolazo)”] adapted from Klandermans (1997). Responses were given on a Likert scale ranging from 1 (never) to 5 (more than five times). A score of five means that respondents never participated and 30 that they did it at maximum. Reliability was satisfactory (α = 0.71).

#### Creativity Responses

##### Cognitive Creativity

Self-reported cognitive creativity was assessed by administering participants three items based on a review of self-reported creativity (de Jong and den Hartog, 2010; da Costa et al., 2015): (a) the number of ideas/proposals that they came up with as a result of the social protests/mobilization process (i.e., fluidity); (b) the extent to which they have applied such new ideas/proposals; and (c) the perceived successful application of the ideas/proposals. Regarding the number of ideas/proposals (i.e., fluidity), responses were given on a Likert scale ranging from 1 (I haven’t come up with any new ideas/proposals) to 4 (I have come up with six or more ideas/proposals). In the case of the application of new ideas/proposals, participants also answered on a 4-point Likert scale (1 = none; 4 = I have set out three or more of my new ideas/proposals). Concerning ideas/proposals’ perceived successful application, a 1–4 response format was used (1 = I have not been successful regarding my new ideas/proposals; 4 = I have been successful in at least one of my new ideas/proposals). Reliability was acceptable (α = 0.67).

In addition to the foregoing, two open questions asked respondents to describe (a) ideas/proposals of different Chilean members or collectives for channeling the situation of the country (“Different members and collectives are generating ideas/proposals to address the situation in their country the situation that your country is going through. Write the one you consider most salient.”) and (b) their personal ideas/proposals concerning the same issue [“Write an idea/proposal (yours) that you consider could contribute to *channeling* the current situation in your country”]. Six blind judges, experts, and novices evaluated, on a scale from 1 (low) to 7 (high), the originality (CCI = 0.67) and efficacy (CCI = 0.82) of participants’ ideas/proposals (total CCI = 0.77). Participants’ originality and efficacy levels were found to be significantly correlated (*r* = 0.51, *p* < 0.001). We calculated an index of creativity by the multiplication of originality and efficacy, thereby reproducing the concept of creativity as innovative and useful ideas/proposals.

#### Social Representations of Demonstrations

Five open questions were asked about the images and opinions of the demonstrators and the police to obtain the beliefs linked to the social representations of the mass movement: (1) Please write three words (terms, adjectives, expressions, etc.) with which you associate the concept minority; (2) Please write three words (terms, adjectives, expressions, etc.) with which you associate the concept “majority”; (3) What images come to mind as most representative of law enforcement (police, military, other)?; (4) What images come to mind as most representative of the protesters’ performance?; and (5) Of what event/experience do the September/October protests/manifestations in your country remind you? All the words were analyzed using Iramuteq.

#### Sociodemographic and Ideological Variables

Age, gender (1 = men, 2 = women), and educational level (1 = basic education; 2 = secondary education; 3 = technique completed; 4 = university completed; 5 = post degree finished) were also included in the survey. Finally, participants’ political ideology was evaluated on a scale ranging from 1 (left wing) to 7 (right wing).

## Results

### Means Comparisons Between Participants and Non-participants

To describe means and examine factors related to participation, individuals who participated in the demonstrations were compared with non-participants. As Table 1 shows, all studied variables (with the exception of gender) reflected differences as a function of participation (vs. non-participation) in the Chilean protests. With respect to demographics, participants were younger, relatively more left-wing, and of higher educational level. As can be seen in Table 1, our results also yielded differences between participants and non-participants in all socio-cognitive indicators, perceived emotional synchrony, collective action measures, and creativity responses.

**TABLE 1:** Mean scores and standard deviations on the various outcomes for the total sample and as a function of individuals’ participation vs. non-participation in the Chilean protests.

	Total sample *N* = 262	Participants *N* = 186	Non-Participants *N* = 76		
		
	*M (SD)*	*M (SD)*	*M* (*SD*)	*df*	*F*
Identification with demonstrators	5.45 (2.05)	6.18 (1.24)	3.68 (2.53)	1	114.90***
Identification with the government	1.16 (1.67)	0.77 (1.32)	2.09 (2.05)	1	38.38***
Index of identification with mobilizations	4.30 (3.35)	5.41 (2.17)	1.59 (4.13)	1	95.07***
Agreement with grievances	3.87 (0.97)	4.20 (0.68)	3.09 (1.13)	1	93.57***
Collective efficacy	4.27 (0.87)	4.40 (0.80)	3.98 (0.98)	1	12.81***
Perceived emotional synchrony	5.10 (1.65)	5.59 (1.35)	3.93 (1.75)	1	68.18***
Repertoire of participation	2.89 (1.44)	3.67 (0.93)	1.00 (0.00)	1	620.14***
Cognitive creativity	2.10 (0.84)	2.17 (0.86)	1.94 (0.79)	1	4.03*
Originality hetero-evaluation	3.98 (0.92)	4.09 (0.89)	3.70 (0.94)	1	9.28**
Efficacy hetero-evaluation	4.13 (1.20)	4.24 (1.15)	3.85 (1.28)	1	5.21*
Judges’ evaluation (originality*efficacy)	16.98 (6.71)	17.80 (6.57)	15.00 (6.69)	1	8.67**
Age	35.99 (12.51)	34.78 (12.13)	38.85 (13.02)	1	5.38*
Political position	3.54 (1.06)	3.31 (0.98)	4.08 (1.08)	1	28.75**
Educational attainment	4.25 (0.60)	4.31 (0.52)	4.10 (0.72)	1	6.36*

N = 262. *p = 0.05, **p = 0.01, ***p ≤ 0.001.

### Correlations of Participation in Demonstrations With Collective Identity Indicators, Collective Effervescence, and Collective Action Measures

To contrast H1, point biserial correlations for the participation in demonstrations variable were utilized (only reported in the text). Participation in demonstrations was positively related to identification with demonstrators (*r* = 0.55, *p* < 0.001), negatively associated with identification with the government (*r* = –0.36, *p* < 0.001), and positively associated with agreement with the protesters’ grievances (*r* = 0.51, *p* < 0.001). Furthermore, participation in demonstrations was positively correlated with collective efficacy (*r* = 0.22, *p* < 0.001), perceived emotional synchrony (*r* = 0.46, *p* < 0.001), and with the repertoire of forms of collective action (*r* = 0.84, *p* < 0.001).

Table 2 displays correlations of collective identity factors and perceived emotional synchrony with collective action variables and creativity indicators.

**TABLE 2:** Correlations of collective identity factors and perceived emotional synchrony with collective action variables and creativity indicators.

	*Collective action indicator*	*Creativity measures*	
	
	Repertoire of participation	Cognitive creativity	Judges’ evaluations
*Collective identity factors*			
Identification with demonstrators	0.57***	0.17**	0.22**
Identification with the government	–0.45***	–0.14*	–0.14*
Index of identification with mobilizations	0.57***	0.17**	0.20**
Agreement with protesters’ grievances	0.56***	0.17**	0.20**
Collective efficacy	0.25***	0.16**	0.18**
*Collective effervescence*			
Perceived emotional synchrony	0.53***	0.20***	0.20**

N = 262. *p ≤ 0.05, **p ≤ 0.01, ***p ≤ 0.001.

### Multiple Regression Analyses Predicting Collective Action Indicators by Collective Identity-Related Variables and Collective Effervescence

A set of multiple regression analyses were computed to test H2. Table 3 gives the findings from the multiple regression analysis predicting collective action-related indicators (i.e., repertoire of forms of collective action) using collective identity-related variables and perceived emotional synchrony as predictors. Sociodemographic and ideological factors (i.e., gender, age, educational attainment, and political orientation) were also included in the regression equation to control for their potential influence.

**TABLE 3:** Multiple regression analyses predicting collective action indicator by collective identity-related variables and collective effervescence.

			Repertoire of participation		
*Predictors*		*b*	β	*95%IC[LL, LU]*	*t*
Age		–0.006	–0.04	[–0.014,0.002]	–1.40
Gender		–0.241	–0.07*	[–0.455, –0.026]	–2.20
Educational attainment		–0.002	–0.01	[–0.168,0.165]	–0.01
Political ideology		–0.064	–0.04	[–0.177,0.050]	–1.10
Participation in demonstrations		2.301	0.72***	[2.04, 2.56]	17.51
Index of identification with mobilizations		0.031	0.07	[–0.011,0.073]	1.44
Agreement with protesters’ grievances		0.069	0.09**	[0.022,0.116]	2.89
Collective efficacy		–0.010	–0.01	[–0.129,0.108]	–0.17
Perceived emotional synchrony		0.088	0.10**	[0.014,0.163]	2.34
Total *R*	0.869				
Total *R*^2^	0.75***				
*F*	76.034***				
Cohen’s *f*^2^	0.25				

*N = 262.*

*Gender: 1 = females, 2 = males. Participation in demonstrations: 2 = yes, 1 = no. b represents unstandardized regression weights. β indicates the standardized regression weights. LL and LU indicate the lower and upper limits of a confidence interval, respectively. All variance inflation factors (VIFs) ≤ 3.45.*

*p ≤ 0.05, **p ≤ 0.01, ***p ≤ 0.001.

Our results indicated that perceived emotional synchrony significantly predicted respondents’ engagement in a rich repertoire of forms of participation in the social movement (β = 0.10, *t* = 2.34, *p* = 0.020).

### Multiple Regression Analyses and Mediational Analyses Predicting Creativity Indexes by Collective Effervescence

Scores of judge’s evaluations of originality and efficacy of ideas written by participants were around the mean (mean 4 and 4.12 or 50 and 52% respectively of the measuring range 1 low to 7 high).^7^ The personal proposals that were used as indicators of cognitive creativity emphasized social reforms 48% (e.g., abolition of the private pension system, wage improvements, free education), social changes 26% (e.g., reorganization of the militarized police, constituent assembly), and improvement of social cohesion 17% (e.g., building of social trust). Destructive proposals like aggression to police or destruction of buildings and monuments were residual.

To test H3, creativity variables (i.e., cognitive creativity and judges’ evaluation of creativity) were regressed on perceived emotional synchrony. Sociodemographic and ideological characteristics (i.e., gender, age, education, and political ideology) were also incorporated as control variables. Self-reported cognitive creativity was positively correlated with judges’ evaluation of creativity (*r* = 0.17, *p* = 0.006). Perceived Emotional synchrony showed similar correlations with creativity indexes (see Table 2).

Consistent with our expectations, multiple regression analysis (see Table 4) revealed that perceived emotional synchrony was a significant predictor of both self-reported cognitive creativity (β = 0.20, *t* = 2.83, *p* = 0.005) and judges’ evaluation of the creativity of respondents’ proposals (β = 0.20, *t* = 2.80, *p* = 0.005).

**TABLE 4:** Multiple regression analyses predicting creativity indexes by collective effervescence.

			Cognitive creativity					Judges’ evaluations (originality*efficacy)		
*Predictors*		*b*	β	*95%IC [LL, LU]*	*t*		*b*	β	*95%IC [LL, LU]*	*t*
										
Age		0.001	0.01	[–0.008,0.010]	0.24		–0.004	–0.008	[–0.078,0.069]	–0.11
Gender		0.155	0.08	[–0.083,0.394]	1.28		0.703	0.05	[–1.20, 2.61]	0.72
Educational attainment		0.078	0.05	[–0.108,0.263]	0.82		0.595	0.05	[–0.877, 2.06]	0.79
Political ideology		–0.095	–0.11 +	[–0.206,0.016]	–1.67		–0.421	–0.06	[–1.29,0.455]	–0.94
Perceived emotional synchrony		0.103	0.20**	[0.031,0.174]	2.83		0.815	0.20**	[0.243, 1.38]	2.80
Total *R*	0.263					0.232				
Total *R*^2^	0.05*					0.05*				
*F*	3.372**					2.502				
Cohen’s *f*^2^	0.93					0.94				
(1.23.										

*N = 262.*

*Gender: 1 = females, 2 = males. b represents unstandardized regression weights. β indicates the standardized regression weights. LL and LU indicate the lower and upper limits of a confidence interval, respectively. All variance inflation factors (VIFs) ≤ 1.23.*

+p ≤ 0.10, *p ≤ 0.05, **p ≤ 0.01.

**FIGURE 4:**
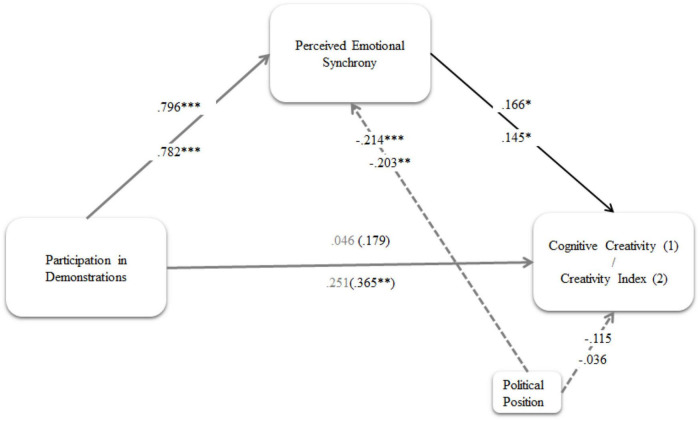
Mediation models H3b: Cognitive Creativity (1) and Creativity Index (2). Standardized indirect effect on: Cognitive Creativity: 0.132 (0.066) [0.013;0.273] on Creativity Index 0.113 (0.058) [0.010;0.240]. Coefficients in the upper side refers to cognitive creativity and in the lower part to creativity index. Standardized regression coefficients are presented. Numbers in parentheses refer to total effect. *p < 0.05, ^**^p < 0.01, ^***^p < 0.001.

Then, a mediation model was performed to examine the second part of H3, applying Hayes’ model 4, and using participation in demonstrations as a predictive variable, perceived emotional synchrony as a mediator, and creativity variables (cognitive creativity and creativity index) as outcomes. Perceived emotional synchrony was significantly associated with both creativity variables (see Figure 4).

Controlling for political position, participation in demonstrations predicted perceived emotional synchrony with a significant coefficient. Higher levels of perceived emotional synchrony were indicative of greater scores on cognitive creativity (0.166) and creativity index (0.145). Political orientation was also a predictor of perceived emotional synchrony. Participation in demonstrations showed a significant direct effect on the creativity index, albeit no significant total effects on cognitive creativity or creativity index emerged.

Moreover, all total indirect effects emerged as significant because the 0 value was not included in any of the CIs generated. Our results revealed that the effect of participation in demonstrations on both measures of cognitive creativity [CI = 0.132 (0.066) (0.013;0.273)] and creativity index [CI = 0.113 (0.058) (0.010;0.240)] were driven by increased perceived emotional synchrony, thus confirming its mediating role.

### Automatic Lexical Analysis of Open Questions and Association With Participation, Social Identification, Collective Efficacy, and Perceived Emotional Synchrony

To examine H4, we used Iramuteq to analyze the words associated with demonstrators, police, majority, minority, and the collective and personal past lived events evoked by demonstrations. We also include quartiles of scales of participation in demonstrations and perceived emotional synchrony, tertiles of identification with demonstrators, collective efficacy, and political position (right, center, or left) as passive variables. We decided to divide the scores into quartiles or tertiles according to their dispersion and ensure the existence of groups of similar size.

Three clusters were found. Table 5 shows the list of words for each cluster, and Table 6 shows the passive variables associated with them. The first lexical class gathered the words justice, right, abuse, injustice, inequality, struggle, repression, bravery, dignity, hope, and unity and was significantly associated with higher levels of participation in the mobilizations, a high tertile of identification with the protesters, higher collective efficacy, perceived emotional synchrony, and being leftist. This cluster emphasizes the struggle for justice and opposing injustice, a positive view of demonstrators, stress repressive reactions, and hope.

**TABLE 5:** Words included in lexical classes.

Words	Cluster 1	Cluster 2	Cluster 3
	Chi-square	Chi-square	Chi-square
Justice	36.051**	−6.372*	−15.099**
Abuses	21.508**	–2.526	−11.199**
Rights	20.805**	−9.21**	–3.543
Fight	20.524**	−10.207**	–2.879
Repression	17.129**	–1.263	−10.682**
Injustice	16.777**	−8.465*	–2.292
Inequality	10.171**	–2.342	–3.544
Brave	9.295*	–1.287	−4.469*
Dignity	9.223*	–1.342	−4.32*
Torture	8.389*	–2.003	–2.84
Hope	7.802*	–1.044	–3.817
Unity	4.185*	3.273	−15.051**
Change	–2.313	19.204**	−6.756*
Military	–2.137	11.643**	–3.001
Demonstration	−6.846*	6.555*	0.087
Movement (social)	–3.563	7.992*	–0.526
Bring (people together)	–1.267	7.179*	–1.914
Pinochet (dictator)	0	5.514*	−4.992*
Penguin (2000 students movement)	–0.801	9.349**	−3.951*
Voice (express claim)	−6.114*	4.777*	0.253
Order	−14.948**	−8.492*	46.901**
Disorder	−12.496**	−7.1*	39.209**
Fear	−12.496**	1.703	6.057*
Security	−4.877*	–2.771	15.304**
Looting	−4.688*	−4.447*	18.419**
Destruction	−5.395*	−4.877*	20.725**
Vandalism	–1.425	–2.363	7.425**
War	−4.159*	–0.372	7.425*

*P < 0.05, **p < 0.01.

**TABLE 6:** Variables associated with the clusters.

	Cluster 1	Cluster 2	Cluster 3
	Chi-square	Chi-square	Chi-square
High quartile participation	12.98**	–0.01	−10.22**
Low quartile participation	−22.01**	0.01	23.1**
High tertile identification demonstrators	30.75**	–0.427	−26.988**
Low tertile identification demonstrators	−34.01**	0.03	39.9**
High tertile collective efficacy	3.98*	0.01	−5.07*
High quartile Perceived emotional synchrony	10.9**	–3.1	–3.1
Low quartile Perceived emotional synchrony	−17.058**	–0.07	21.053**
Left	15.74**	–1.004	−10.273**
Center	–0.211	5.354*	–3.005
Right	−16.525**	−5.495*	42.399**

*p < 0.05, **p < 0.01.

The second lexical class includes words such as change, social movement, demonstration, voice (give voice, claim), Pinochet, military, secondary school 2000s demonstrations, and was associated with a centrist political position. This cluster suggests a mixed representation of mass movement, including positive attributes but also remembering past failed social movements and the threat of military putsch.

The third lexical class includes words such as order, disorder, security, fear, vandalism, destruction, looting or riots, and war. This cluster was associated with low identification with demonstrators, being rightist, and non-participation in demonstrations. This cluster stresses threats to order, negative emotions, and collective behaviors and emphasizes the need of order and security.

## Discussion

The present study aimed at exploring the psychosocial factors at work in the Chilean protests of 2019--2020^8^. Overall, we found that participants, compared with non-participants, reported higher identification with demonstrators (and lower identification with the government), described a greater level of agreement with protesters’ grievances, showed higher perceived emotional synchrony, were engaged in more forms of collective action, and expressed greater creativity in their responses. Social identification with the social movement also predicts participation in a richer repertoire of collective action (Drury, 2014). Supporting our second hypothesis, perceived emotional synchrony significantly predicted agreement with a large repertoire of collective actions and beliefs linked to the movement (see below). In this sense, it is plausible to sustain that the socio-emotional path is at least as relevant as the socio-cognitive path to collective action. Perceived emotional synchrony as a state of shared high emotional arousal “reinvigorates” subjects and fuels their orientation toward changing the social milieu (Durkheim, 1912/2001; Páez et al., 2015).

In addition, we showed that, even when controlling for several sociodemographic and ideological factors, perceived emotional synchrony predicted self-reported cognitive creativity and judges’ evaluation of the creativity of participants’ proposals. In addition, mediational analyses confirmed that collective effervescence, as assessed by the PES (Perceived Emotional Synchrony) scale, played a mediation role between participation in demonstrations and creativity. Self-reported creativity showed higher explained variance that hetero-evaluated creativity of participants’ proposals. This is an important point because creativity was not only self but also hetero-evaluated, adding validity to our measures. Effect sizes were lower in comparison to self-reported behaviors and beliefs, but in the rank of association of this type of measure of creativity (*r* = 0.20) (da Costa et al., 2015; Gajda et al., 2017). This is a relevant result because it confirms Durkheim’s idea that feelings of collective effervescence reinforce cognitive creativity at an individual level. This means an effervescent emotional atmosphere facilitates the experience of novel and useful cognitive products. How can collective effervescence or emotional synchrony explain political creativity? The convergence and intensification of emotions will fuel high levels of positive personal and collective emotions, particularly of the transcendence of the self (Wlodarczyk et al., 2020; Zumeta et al., 2020). As we argue in the introduction, high arousal positive affect is related to creativity by enhanced availability of memory, global attention, and a more holistic, flexible, and integrative way of thinking. This intensified level of positive valanced emotions will facilitate the accessibility of positive political memories, the broadening of attention, the flexibility of political thought and behavior, and as Fredrickson (2013) states, will broaden the repertoire of political behaviors and ideas. Moreover, the affective content of the collective effervescence on Chilean 2019 demonstrations includes hope, solidarity, elevation, or moral inspiration, feeling moved by love, and social awe. These self-transcendent emotions focus on others, based on shifting attention toward the needs and concerns of others. They made the collective self more salient, reinforcing meaning in life and a more benevolent view of the social world (Van Cappellen and Rimé, 2014). This will lead to increased creativity regarding the collective good and the social world, facilitating creativity in the socio-political area.

Creativity means that cognitive products are new and helpful. Of course, creativity can have a negative facet because new ideas and behavior can be destructive. Dark creativity is defined as the use of new ideas and behaviors to gain an unfair advantage or to deliberately damage others (Cropley, 2011). Limited research shows that creative individuals tend to be more dishonest, probably because they share a heightened feeling of being unconstrained by rules (Gino and Wiltermuth, 2014). Regarding personality, creativity was related to facets of the Dark personality triad, like narcissism and reduced reality testing, but was negatively correlated with Machiavellianism and subclinical psychopathy, while it was positively associated with openness and extraversion. These results suggest that the bright side of personality seems to have a much closer link to creativity than dark personality traits do (Dahmen-Wassenberg et al., 2016). The vandalization of monuments and the buildings destroyed by fire could be conceived as a type of dark creativity that characterized Chilean social unrest, as a reviewer suggested. However, the content of proposals and ideas was overwhelmingly positive, as we saw in the results. Globally, dark creativity is not a salient process in this study. Of course, the novelty and, above all, the functionality or usefulness of the ideas depended on a framework of judgment. In this case, the evaluations judged the extent to which the proposed ideas could serve for social change. From a frame of judgment that the most functional is to maintain the *status quo*, these ideas would be evaluated as dysfunction.

Finally, three sets of beliefs were found by automatic lexical analysis. The first set or lexical cluster found by Iramuteq could be conceived of as a positive romantic view of popular masses, or in Durkheim’s view, social representations of demonstrators as “just fighters in reaction to injustice and inequality.” This social representation was associated with a high level of participation and with factors conducive to higher identification, perceived emotional synchrony, and to high positive moral emotions such as bravery, dignity, and hope, which were words included in this cluster. This set of beliefs is akin to a positive view of populist movements and to a “Durkheimian” view that posits collective gatherings enhance social cohesion and moralization along with renewed agreement upon values (Rimé et al., 2020). For Durkheim (1995/1912), values and symbols are created and invented in the midst of this collective effervescence (Moscovici, 1988). Interviews with participants support this set of beliefs because they reported they were positively surprised by the great social mobilization, felt inspired by the “first line fighters,” and felt the large demonstrations and protests recreated a sense of community (Claude, 2020; Efe/El Mostrador Braga, 2020). The second set also perceives mass movements as related to change, giving voice to claims as a set of demonstrations, but was anchored in past failures and repression (Pinochet, military threat). This ambivalent social representation was associated with the political center, but not with psychosocial variables. The third set of beliefs represents a last minoritarian point of view and could be interpreted as a social representation of populists’ social movements as “barbarian disorder.” These beliefs reproduce Lebon’s negative views of mass and crowd psychology as mob mentality and are related to a negative attitude toward the social movement and to the political right. This “Lebonian” view of crowds, as mobs emphasizes negative aspects of mass movements (Moscovici, 1988), legitimizes interventions of social control by the state to restore order, and is akin to a negative view of populism as a danger to social order and democracy (Noury and Roland, 2020).

### Limitations

Although the present research provided new insights into the relationship between emotions, social representations, and collective action and confirmed the empowering role of emotional synchrony during collective gatherings (Wlodarczyk et al., 2020), it was not devoid of limitations. The first and main limitation relates to its cross-sectional design, which prevented testing for causal relationships. This was mainly due to the specificities of the context (i.e., a large-scale spontaneous protest) and to our willingness to favor ecological validity over controllability. Ideally, future studies should address this limitation by using longitudinal designs. A second limitation pertains to the specificities of the sample, which was composed of students from private universities located in big cities. We acknowledge that a higher number of participants would be ideal, however, due to the context in which we collected the information and the urgency of collection within the time of the protests, the number of participants was the maximum possible. While students formed an important part of the protest movement in Chile, it encompassed a much larger part of the population and a greater variety of ages. Even though this sample could be more socially engaged than other groups, previous research shows that young people do not have a marked or homogeneous political position and that most of them do not position themselves politically on the left or the right, although 48% do consider themselves to be opposed to the government (Feedback, 2019). Third, the use of self-reported questions and shared method variance could explain part of the associations found. However, we also used hetero-evaluation for creativity, and results were similar to self-reported creativity measures. Fourth, the use of two single items for measuring social identification, due in part to the length of the questionnaire, may appear insufficient. Finally, although Iramuteq generates “objectively” lexical classes, a degree of interpretation is necessary to categorize them as types of social representation. However, the ways in which respondents communicate about the social conflict in their context was not examined because it would have required at least focus groups of demonstrators and systematic observation. More attention should be paid to these aspects in future studies.

## Conclusion

Our results confirmed the idea of a strong politicized identity, where several factors of collective identity such as social identification with demonstrators, misidentification with the elite and the government, agreement with protesters, and collective efficacy, but also socio-emotional aspects of perceived emotional synchrony seem to be closely related to participation in a populist social movement. Results corroborate that at an individual level, feelings of collective effervescence reinforce cognitive creativity (Durkheim, 1912/2001). In this regard, the theoretical path of integrating socio-cognitive aspects into a neo-Durkheimian framework of collective effervescence proves useful for sociopolitical events of great importance, such as the Chilean case. Finally, a set of beliefs related to injustice, fighting oppression, bravery, and hope characterizes positive social representations of populist mass movement.

## Data Availability Statement

The datasets presented in this study can be found in online repositories. The names of the repository/repositories and accession number(s) can be found below: The datasets and survey material supporting this article have been uploaded on the Open Science Framework: https://osf.io/csn7u/?view_only=8c97ad223d22499ab67596693059a3fb.

## Ethics Statement

The studies involving human participants were reviewed and approved by the University of the Basque Country. The data recorded was alphanumerically code to ensure anonymity following the Organic Law on the Protection of Personal Data (BOE-A-2018-16673), and compliance with the regulation of the Ethics Committee for Research Involving Human Beings (CEISH) by the University of the Basque Country. The patients/participants provided their written informed consent to participate in this study.

## Author Contributions

DP, PC-A, SD, AC-M, MG-L, PB, and GN-C: conceptualization. DP, PB, and GN-C: methodology. GN-C, PC-A, MG-L, AC-M, PB, and DP: formal analysis and investigation. DP, PC-A, AC-M, and GN-C: writing – original draft preparation. DP, SD, MG-L, and LM: questionnaire preparation. All the authors read and approved the final manuscript for submission.

## Conflict of Interest

The authors declare that the research was conducted in the absence of any commercial or financial relationships that could be construed as a potential conflict of interest.

## Publisher’s Note

All claims expressed in this article are solely those of the authors and do not necessarily represent those of their affiliated organizations, or those of the publisher, the editors and the reviewers. Any product that may be evaluated in this article, or claim that may be made by its manufacturer, is not guaranteed or endorsed by the publisher.

## Footnotes

1 Features like the homogeneity of the people, its exclusivity, greater direct rule, nationalism, a single leader embodying the people, antagonism to vulnerable out-groups, and supposed people’s innate wisdoms, demands for direct action and rejection of deliberations to democratically agree upon decisions, are frequently but not always associated to populism – and are these characteristics that often represent dangers to tolerance and social inclusion (Mansbridge and Macedo, 2019). Moreover, studies on populist beliefs and attitudes found that anti-elitism, preference for popular sovereignty, a belief in the homogeneity and virtuousness of the people, Manichaean or dichotomist polarized views, and nationalism are not strongly associated, show different correlates and predictive values regarding populist parties’ support (Schulz et al., 2017; Castanho Silva et al., 2019; Van Hauwaert et al., 2019).

2 The current operationalization of collective effervescence in social psychology.

3 As an illustration, the results of a youth-focused national survey conducted in November and December 2019 (Feedback, 2019) indicated that the percentage of people interested in talking about politics with friends and family and seeing news about political issues in Chile increased, respectively, from 30 and 24% in 2018 to 45 and 43% in 2019. In parallel, the level of interactions on social media increased significantly. In fact, young people trust social media 52% (e.g., Facebook, Instagram) more than traditional mass media such as TV (9%) or radio (8%). As a result, they are mostly informed about demonstrations through social media, more than any other source of information (Feedback, 2019).

4 A third support “thought” demonstrations: 37% support the use of barricades, 35% face the police, and 19% support the burning of buildings (Feedback, 2019).

5 A majority of the Chilean population justifies and supports the social protest—90, 83, and 72% in three representative surveys in October, and still 80, 66, and 59% in 2020 (Activa, 2020; Cadem, 2020; Criteria, 2020). Mobilizations are mostly perceived as an expression of social discontent (73%), although a minority believes they are a problem of public order and organized violence (16.5%, Cadem, 2020). Brutal repression by the police and security forces caused about 40 deaths, 200 eye injuries from anti-riot bullets, and 1,400 casualties by gunshot. Around 10,000 individuals have been jailed (Instituto Nacional de Derechos Humanos, n.d.). Surveys show that trust in police decreased from 57% in 2009 to 38% in 2016, 23% in 2018, and 10% in 2020 (Feedback, 2019).

6 The sample size was calculated using the software G*Power (Faul et al., 2007), for a multiple linear regression predicting the dependent variables *a priori*: two-tailed, with an effect size (f2) = 0.15, α = 0.01, power = 0.90, and accounting for 9 predictors = 9, the analysis showed a resulting sampling of 89 participants.

7 A content analysis and a lexical analysis with Iramuteq of ideas could be useful, as a reviewer suggested, but we lack the space to carry out this analysis.

8 At the descriptive level, our results are congruent with those of general surveys (Cadem, 2020; Feedback, 2019; Activa, 2020; Criteria, 2020). The majority of participants reported high identification with demonstrators, rejected identification with government, and were characterized by a medium level of participation in demonstrations. Agreement with the protesters’ grievances was high, as well as perceived collective efficacy and levels of collective effervescence or perceived emotional synchrony. Self-reported creativity and judges’ evaluation of the creativity of participants’ proposals were medium. This profile suggests a situation of strong politicized identity and collective effervescence, with more importance of emotional than cognitive creative reactions.
